# Supplementary material for: Multi‐dataset identification of innovative feature genes and molecular mechanisms in keratoconus
Source: J Cell Mol Med. 2024 Sep 19;28(18):e70079. doi: 10.1111/jcmm.70079 (PMC11412914; doi:10.1111/jcmm.70079)
Supplement: Supplementary file 2 — Material S2. [file JCMM-28-e70079-s002.zip › 7.GSEA analysis/5.RNF217-AS1/KEGG_THYROID_CANCER.html]

Details for gene set KEGG\_THYROID\_CANCER[GSEA]

|  || Dataset | RNF217-AS1.group.cls#h\_versus\_l |
| Phenotype | group.cls#h\_versus\_l |
| Upregulated in class | l |
| GeneSet | KEGG\_THYROID\_CANCER |
| Enrichment Score (ES) | -0.40501052 |
| Normalized Enrichment Score (NES) | -1.4036642 |
| Nominal p-value | 0.0734127 |
| FDR q-value | 0.35937208 |
| FWER p-Value | 0.864 |
Table: GSEA Results Summary

  

Fig 1: Enrichment plot: KEGG\_THYROID\_CANCER      
 Profile of the Running ES Score & Positions of GeneSet Members on the Rank Ordered List

  

| SYMBOL | TITLE | RANK IN GENE LIST | RANK METRIC SCORE | RUNNING ES | CORE ENRICHMENT || 1 | BRAF | na | 5517 | 0.199 | -0.0879 | No |
| 2 | PPARG | na | 5598 | 0.196 | -0.0204 | No |
| 3 | NTRK1 | na | 9718 | 0.091 | -0.1065 | No |
| 4 | KRAS | na | 11445 | 0.064 | -0.1335 | No |
| 5 | CDH1 | na | 11891 | 0.057 | -0.1260 | No |
| 6 | RET | na | 12518 | 0.048 | -0.1268 | No |
| 7 | TPM3 | na | 12575 | 0.048 | -0.1113 | No |
| 8 | TCF7L2 | na | 12940 | 0.043 | -0.1065 | No |
| 9 | MAPK1 | na | 13362 | 0.038 | -0.1051 | No |
| 10 | MAPK3 | na | 14127 | 0.029 | -0.1167 | No |
| 11 | HRAS | na | 15878 | 0.010 | -0.1635 | No |
| 12 | NCOA4 | na | 16052 | 0.008 | -0.1656 | No |
| 13 | MAP2K2 | na | 16236 | 0.006 | -0.1686 | No |
| 14 | TCF7L1 | na | 16424 | 0.004 | -0.1724 | No |
| 15 | RXRA | na | 16489 | 0.004 | -0.1729 | No |
| 16 | NRAS | na | 17304 | -0.005 | -0.1947 | No |
| 17 | TFG | na | 17759 | -0.009 | -0.2047 | No |
| 18 | CCND1 | na | 18820 | -0.019 | -0.2286 | No |
| 19 | CTNNB1 | na | 19818 | -0.028 | -0.2473 | No |
| 20 | TCF7 | na | 19939 | -0.029 | -0.2403 | No |
| 21 | CCDC6 | na | 20543 | -0.035 | -0.2450 | No |
| 22 | MAP2K1 | na | 23215 | -0.064 | -0.2991 | No |
| 23 | RXRB | na | 24704 | -0.086 | -0.3113 | No |
| 24 | PAX8 | na | 25526 | -0.099 | -0.2996 | No |
| 25 | TPR | na | 27152 | -0.129 | -0.3004 | No |
| 26 | MYC | na | 30784 | -0.234 | -0.3216 | Yes |
| 27 | TP53 | na | 32227 | -0.304 | -0.2545 | Yes |
| 28 | RXRG | na | 33670 | -0.428 | -0.1432 | Yes |
| 29 | LEF1 | na | 34024 | -0.487 | 0.0203 | Yes |
Table: GSEA details [plain text format]

  

Fig 2: KEGG\_THYROID\_CANCER      
 Blue-Pink O' Gram in the Space of the Analyzed GeneSet

  

Fig 3: KEGG\_THYROID\_CANCER: Random ES distribution      
 Gene set null distribution of ES for **KEGG\_THYROID\_CANCER**
